# Correlative SEM–AFM Characterization of 2D Materials: From Sequential Measurements to In-Situ Multimodal Platforms

**DOI:** 10.3390/nano16191199

**Published:** 2026-09-22

**Authors:** Xuan Wu, Wenping Li, Shu Li, Zhe Wang, Wei Cai, Shouzheng Guo, Xunan Ran, Junying Zhang

**Affiliations:** 1 School of Physics, Beihang University, Beijing 100191, China; 2 School of Cyber Science and Technology, Beihang University, Beijing 100191, China

**Keywords:** 2D materials, stimuli-responsive materials, correlative microscopy, SEM–AFM, multimodal characterization, in-situ characterization, AI analysis

## Abstract

With atomic-scale thickness and defect-sensitive structures, stimuli-responsive two-dimensional (2D) materials exhibit diversified and highly localized physicochemical behaviors under external triggers such as electric fields, illumination, mechanical strain and chemical environments. These behaviors are intricately coupled with layer variations, grain boundaries, folds and local strain fields, which makes it necessary to reveal the structure–performance relationships of stimuli-responsive 2D materials. Compared with conventional single-technique characterization, the combination of scanning electron microscopy (SEM) and atomic force microscopy (AFM) provides a powerful framework for correlating structural, morphological and functional information across length scales. In this review, a systematic overview of correlative and integrated SEM–AFM characterization of 2D materials is given, covering its origins, working principles, typical applications and recent progress. Representative measurement configurations are classified into three categories—sequential SEM and AFM characterization, integrated AFM-in-SEM platforms, and truly in-situ or simultaneous SEM–AFM measurements—and the corresponding applications to stimuli-responsive 2D materials are discussed with respect to electrical, optical, mechanical and multiphysics characterization. The experimental challenges and limitations of SEM–AFM integration, including electron-beam effects, contamination, vacuum constraints and drift-related issues, are critically analyzed. Finally, the development of intelligent SEM–AFM platforms accompanied by emerging deep learning and artificial intelligence (AI) paradigms is outlined as a perspective, with emphasis on multimodal data fusion and automated feature extraction.

## 1. Challenges in 2D Materials Driving the Development of Multimodal Characterization Techniques

### 1.1. 2D Materials: Structure, Stimuli-Responsiveness, and Significance

2D materials are low-dimensional systems confined to atomic or quasi-atomic thickness along one spatial dimension while maintaining periodic in-plane structures. As shown in Figure 1, 2D layered materials are characterized by strong intralayer covalent or ionic bonding and weak interlayer van der Waals interactions, resulting in highly anisotropic layered structures [1]. The reduced dimensionality and weak interlayer interactions of 2D materials give rise to thickness-dependent electronic, optical, and mechanical properties distinct from those of bulk materials [2]. At the microscopic level, features such as layer number, edges, grain boundaries, point defects, wrinkles, and strain-concentrated regions are commonly exposed and directly influence local electronic states and interfacial interactions. Consequently, external stimuli can be locally amplified around defect regions, resulting in measurable variations in electronic and nanomechanical properties [3].

Since 2004, research on 2D materials has expanded rapidly to encompass a wide range of layered systems, including transition-metal dichalcogenides (TMDs) such as MoS_2_ and WSe_2_, black phosphorus (BP), and MXenes (Figure 1). Early studies revealed that 2D materials exhibit unique responses to external stimuli, particularly mechanical strain, which are fundamentally different from those of their three-dimensional (3D) counterparts, providing a foundation for the design of strain-engineered materials [4]. Layer-number identification in graphene was initially achieved by atomic force microscopy (AFM) via precise height measurements, establishing a benchmark for thickness characterization across 2D systems. In parallel, Raman and photoluminescence (PL) spectroscopy were introduced to probe layer number, strain, and charge carrier behavior. Notably, PL measurements of monolayer MoS_2_ revealed a transition from an indirect to a direct bandgap, providing a canonical example of layer-dependent optical properties [5].

Under external stimuli, 2D materials exhibit diverse and tunable responses. For example, electric fields can modulate charge carrier dynamics and band alignment through charge transfer and band reconstruction processes [6]. Light excitation can induce exciton formation and non-equilibrium charge carrier distributions [7]. Mechanical strain can modify bond lengths and lattice symmetry, thereby tuning the band structure and electronic transport properties [8]. Gas adsorption as a chemical stimulus can significantly modify the surface electronic states of 2D monolayers via adsorption-induced charge redistribution [9]. These responses are often localized at defects, grain boundaries, edges, or interlayer interfaces, with mechanisms varying across material systems. Graphene primarily responds to electric fields and strain, while TMDs exhibit thickness-dependent electronic structures and are sensitive to external environmental perturbations [10]. Furthermore, 2D materials with anisotropic lattices can display direction-dependent responses, while materials with abundant surface functional groups exhibit interfacial modulation under chemical or electrochemical stimulation. Since the isolation of graphene, 2D materials have been extensively explored for their diverse responses to external stimuli and their applications in multifunctional systems [11]. Their atomic-scale thickness, controllable layers, and tunable interfaces offer unique advantages for device miniaturization, high performance, and functional adaptability.

As research progresses toward performance modulation, efforts increasingly focus on the deliberate engineering of stimulus–response behaviors. Strain engineering has been widely used to modulate the optoelectronic response of 2D semiconductors, including TMD-based photodetectors [12]. Meanwhile, the development of heterostructures, such as MoS_2_/WS_2_ systems, enables controllable interlayer charge transfer and band alignment, thereby enhancing the performance of photodetectors [13]. With the development of stimuli-responsive 2D materials in device integration and multifield coupling, their applications have evolved from fundamental sensing to flexible optoelectronics, intelligent sensing systems, and strain-engineered logic devices. For example, MoS_2_/MoTe_2_ heterostructure photodetectors exhibit broadband photoresponse with tunable sensitivity [14], while BP-based van der Waals heterostructures enable strain-induced modulation of electronic properties including band structure and piezoelectric response [15], highlighting the increasing complexity of structure–property relationships in functional 2D systems.

Characterization techniques play an irreplaceable role in 2D materials science and engineering as they are essential for observing microstructures, understanding material properties, and enhancing device functionality. However, for stimuli-responsive 2D materials with complex structures, single-scale or single-property characterization is inadequate because of the complexity of multiphysics response behaviors under external stimuli [16]. To advance stimuli-responsive 2D materials, it is necessary to develop a new in-situ characterization method with high resolution and multimodality to reveal structural, morphological, and functional responses across scales under near-realistic conditions to elucidate response mechanisms under electrical, optical, mechanical and chemical stimuli.

### 1.2. Development of SEM and AFM Characterization Techniques for 2D Materials

#### 1.2.1. Development History of SEM and AFM

Scanning electron microscopy (SEM) originated in the 1930s, when Manfred von Ardenne proposed the concept of raster scanning imaging using a focused electron beam [17]. Subsequent advances in electron sources, electromagnetic lenses, vacuum systems, and detector technologies have continuously improved SEM performance, enabling its widespread application in materials characterization [18,19]. Further improvements in electron source stability and imaging optimization have enhanced SEM resolution and reliability, extending its application toward nanoscale investigations [20]. In the 21st century, SEM has become an essential tool for the characterization of nanostructures, thin films, and low-dimensional materials [21].

Recently, the integration of artificial intelligence (AI) and machine-learning algorithms has further improved the efficiency and reliability of SEM-based analysis. Automated image segmentation, defect recognition, and structural classification can be performed directly on large microscopy datasets, enabling rapid extraction of microstructural information while reducing user-dependent variability [22]. Although ultrathin 2D materials generally exhibit weak and complex SEM contrast, optimized imaging conditions combined with AI-assisted image analysis have enabled more reliable identification of layer number, flake edges, overlapping regions, growth domains, and defect structures, making SEM an important characterization technique for graphene and other 2D materials [23,24].

Although SEM provides high-resolution structural information, its imaging capability is primarily limited to surface projection and may require additional approaches for accurate three-dimensional surface characterization, particularly for non-conductive materials. Scanning probe microscopy (SPM) techniques, especially AFM, were introduced by Binnig et al. in 1986 as an extension beyond the limitations of scanning tunneling microscopy (STM), enabling three-dimensional surface characterization of both conductive and insulating materials [25]. AFM provides localized nanoscale analysis of surface morphology and mechanical properties across a wide range of materials [26]. With the development of multimode scanning probe microscopy, AFM has achieved atomic-scale resolution under carefully controlled environments [27].

Since the isolation of monolayer graphene by Novoselov and Geim in 2004, AFM has become an indispensable technique for the characterization of 2D materials [28]. AFM enables accurate measurement of thickness and surface features, including wrinkles, roughness, and surface reconstruction [29]. In addition, mechanical properties such as friction, elastic modulus, and adhesion can be quantitatively evaluated through force spectroscopy and lateral force microscopy [30]. AFM-based nanomechanical manipulation also provides a method for nanoscale surface cleaning by mechanically removing adsorbates and residues, facilitating high-resolution characterization of graphene and other atomically thin materials [31]. Beyond conventional topographic imaging, multifunctional AFM modes, including conductive AFM (C-AFM) [32] and Kelvin probe force microscopy (KPFM) [33], have further expanded AFM capabilities toward electrical, mechanical, and interfacial property characterization.

Alongside hardware advances, recent developments in AI-assisted analysis have further enhanced the capability of AFM and scanning probe microscopy. Deep-learning approaches have demonstrated strong performance in automated feature recognition, defect identification, image segmentation, and quantitative analysis of topographical and functional datasets. For 2D materials, machine-learning-based identification of exfoliated flakes, thickness variations, and local structural heterogeneities provides an efficient approach for accelerating data interpretation and improving measurement reproducibility [34]. These developments have established the foundation for future multimodal and correlative characterization approaches combining complementary microscopy techniques.

#### 1.2.2. Development of SEM–AFM Correlative Systems

As shown in Figure 2, the development of correlative SEM–AFM microscopy has progressed from early hybrid instruments toward compact and multifunctional AFM-in-SEM platforms. Early studies demonstrated that AFM could be operated directly within or in conjunction with an SEM, combining wide-field electron imaging with localized surface probing. A notable early example was reported by Stahl et al. in 1994 [35], who developed an AFM using self-sensing piezoresistive cantilevers and integrated it with an SEM. The system enabled simultaneous real-time operation of AFM and SEM, demonstrating the feasibility of compact, optically independent cantilever detection for correlative microscopy [35]. Troyon et al. subsequently introduced a combined scanning force microscope–SEM system for multidimensional analysis, enabling correlated electron, topographical, and frictional imaging. Further developments improved probe positioning, system compactness, and vacuum compatibility [36].

Building on these early concepts, self-sensing and self-actuated cantilevers became increasingly important for the development of compact AFM-in-SEM instrumentation. Angelov et al. demonstrated a six-axis AFM-in-SEM platform based on self-sensing cantilevers, while Kreith et al. developed a versatile AFM module directly integrated into an SEM [37]. Rangelow et al. further extended this concept toward correlative microscopy and nanofabrication within combined SEM/FIB environments. More recent platforms have progressively incorporated multimodal surface analysis, nanomanipulation, energy-dispersive X-ray spectroscopy (EDS), and focused ion beam (FIB) capabilities, enabling increasingly comprehensive correlative characterization workflows [38,39,40,41,42,43].

**Figure 2:**
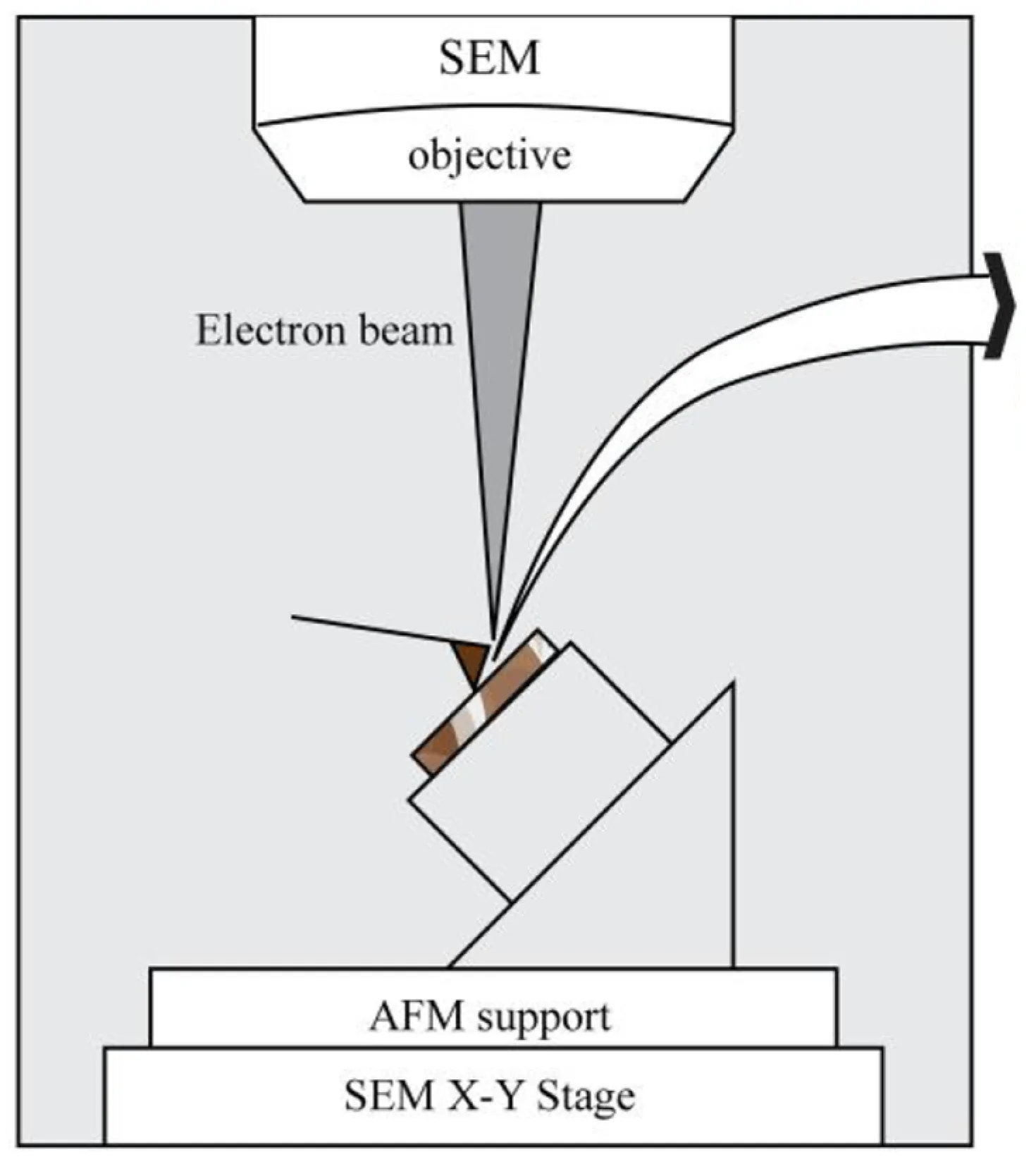
Schematic illustration of the integrated SEM–AFM system. Adapted from Swain et al. [41] under the terms of the Creative Commons Attribution 4.0 International License (CC BY 4.0); images were cropped and relabeled.

A single technique captures only limited aspects of material properties. SEM enables high-throughput, wide-field imaging but may require conductive coatings or charge-control conditions for poorly conductive bulk samples and cannot directly measure mechanical properties [44]. AFM provides high-resolution 3D topography and mechanical mapping but suffers from low throughput and a limited scan area, while lacking intrinsic chemical contrast [45,46]. This complementarity makes it difficult for any single method to fully resolve structure–property relationships in 2D materials. Therefore, the complexity of 2D systems necessitates multiscale, multiphysics correlative approaches. Integrated SEM–AFM platforms address these limitations by enabling comprehensive characterization with minimized risk of damage when electron-beam exposure is carefully controlled (Section 4.1) and have become a key direction in advanced microscopy research.

Conventional AFM systems detect cantilever deflection optically, using a laser beam reflected from the cantilever onto a position-sensitive photodetector; this detection scheme is referred to as optical beam deflection (OBD). Although OBD-based AFM provides high sensitivity and is widely used in ambient and ultrahigh-vacuum (UHV) environments, the OBD geometry is difficult to implement inside the confined SEM chamber: the space required for the laser path and detector assembly conflicts with the electron column and the limited working distance, and the optical components can obstruct electron detection and stage movement. These constraints motivated the development of compact self-sensing cantilevers, in which the deformation or oscillation of the cantilever is detected electrically, eliminating the need for optical alignment [47]. The evolution from OBD-based to self-sensing cantilever designs is therefore a central enabler of SEM–AFM integration, and the two most widely adopted self-sensing mechanisms—piezoresistive cantilevers and A-Probe-based cantilevers—are described in detail in Section 2.1.

### 1.3. Terminology and Classification of SEM–AFM Measurement Configurations

We performed a systematic literature search in the Web of Science and Scopus databases for peer-reviewed studies combining scanning electron microscopy and atomic force microscopy applied to the characterization of two-dimensional (2D) materials, using keyword combinations such as “SEM–AFM”, “AFM-in-SEM”, “correlative AFM/SEM”, “In-situ SEM–AFM”, and “2D materials”. Retrieved records were screened by title and abstract, and the selected studies were classified according to the measurement configurations defined in this section.

Based on the literature search, the multiscale and multiparameter characterization demands of 2D materials have driven SEM–AFM from standalone imaging toward correlative, multimodal, and in-situ systems. Throughout this review, the following terms are used with precise meanings. Multimodal characterization refers to the acquisition of multiple complementary property channels (e.g., topographical, electrical, optical, and mechanical) from the same or related sample regions. Correlative SEM–AFM denotes workflows in which SEM and AFM datasets acquired from the same region of interest are spatially registered and jointly interpreted, regardless of whether the measurements were performed within a single instrument. Integrated SEM–AFM (also referred to as AFM-in-SEM) denotes instrumentation in which the AFM module is physically installed inside the SEM chamber, enabling electron imaging and probe-based measurements within a common vacuum environment. Sequential measurements are performed one after another, typically involving sample transfer between separate instruments, whereas simultaneous (truly in-situ) measurements are performed concurrently on the same sample region, often under an applied external stimulus.

Based on these definitions, representative studies discussed in this review are classified into three categories: (i) sequential SEM and AFM characterization—including SEM-guided workflows and correlative same-region analyses performed sequentially with separate instruments; (ii) integrated AFM-in-SEM platforms; and (iii) truly in-situ or simultaneous SEM–AFM measurements, in which electron imaging and AFM probing are performed concurrently, typically under an applied external stimulus. This classification is used consistently throughout the manuscript, and a summary of representative studies is provided in Section 3.5.

A representative configuration of an integrated SEM–AFM platform is illustrated in Figure 3. In such systems, a compact AFM module is incorporated into the SEM chamber, allowing the AFM probe and electron beam to access the same sample region within a common measurement environment. The coordinated geometry of the SEM, AFM probe, and sample enables direct correlation between electron microscopy and local topographical, mechanical, or functional measurements. Depending on the operating mode, SEM and AFM can be performed sequentially within the same chamber or simultaneously for in-situ characterization. When combined with external stimuli, such platforms further enable direct observation of stimulus-induced structural and functional evolution in 2D materials, as discussed in Section 3.4.

## 2. Fundamentals of SEM–AFM Correlative Microscopy

### 2.1. SEM–AFM Principles and System Architecture

SEM–AFM systems integrate the complementary capabilities of scanning electron microscopy and atomic force microscopy within a single analytical platform. A typical SEM–AFM instrument consists of a miniaturized AFM module installed directly inside the SEM chamber, allowing both the electron beam and AFM probe to access the same region of interest. Due to the limited space and complex operating environment inside the SEM chamber, compact scanners and self-sensing cantilevers are generally preferred. Currently, two representative AFM sensing mechanisms are commonly integrated into SEM–AFM hybrid systems: piezoresistive AFM (Figure 4a) and A-Probe-based AFM (Figure 4b). These approaches employ different electrical detection schemes.

Piezoresistive cantilevers incorporate a doped piezoresistive element, typically a meandering silicon or polysilicon resistor, located near the cantilever base where bending-induced stress is largest. When the cantilever deflects under tip–sample interaction forces, the stress changes the resistance of the piezoresistor; this resistance variation is generally converted into a voltage signal through a Wheatstone bridge readout architecture, in which the cantilever resistor constitutes one active arm and the bridge configuration provides common-mode rejection of temperature drift. Because the deflection signal is purely electrical, piezoresistive cantilevers are compact and can operate in confined environments such as SEM chambers; with optimized designs, noise levels comparable to those of optical beam deflection can be achieved [47].

The A-Probe represents an alternative self-sensing architecture based on active cantilevers, in which both actuation and detection are integrated on-chip. Oscillation of the cantilever is excited electrically by an integrated actuator (e.g., a thermal or piezoelectric element), and the resulting vibration is detected through an integrated sensing element (e.g., a piezoresistive readout or a capacitive/piezoelectric transducer), without any external optical path. This integrated actuation-and-sensing scheme allows dynamic modes (e.g., intermittent-contact imaging) to be operated inside the SEM chamber as easily as static modes, and it is the sensing principle adopted by several commercial AFM-in-SEM modules [47]. These self-sensing approaches eliminate the requirement for optical beam-deflection alignment and provide improved compatibility with the confined environment of SEM chambers.

Despite these advantages, self-sensing cantilevers also have several important limitations. First, probe cost and availability: self-sensing cantilevers are more expensive and are offered by fewer suppliers than conventional OBD cantilevers, and the available range of tip materials, spring constants, and coatings is more limited, reducing experimental flexibility. Second, signal-to-noise ratio: although state-of-the-art piezoresistive cantilevers approach the noise performance of OBD systems, the integrated sensing elements generally exhibit higher noise and lower bandwidth than OBD detection [48], with consequences for weak signals and very small features that are discussed further in Section 4.3. Third, force sensitivity and calibration: the deflection sensitivity of self-sensing cantilevers is device-dependent and may drift, requiring careful calibration, whereas OBD systems can be calibrated geometrically. These trade-offs are particularly relevant for atomically thin 2D materials, where gentle and quantitative force control is essential (Section 4.4).

The operating principle of SEM–AFM relies on the complementary strengths of the two techniques [37]. SEM provides rapid large-area imaging and efficient navigation of regions containing structural features such as grain boundaries, wrinkles, folds, cracks, and thickness variations. AFM subsequently performs localized nanoscale measurements with sub-nanometer vertical resolution, enabling quantitative analysis of surface roughness, layer thickness, edge morphology, and defect structures. Through accurate coordinate correlation, SEM images can be directly linked with AFM measurements acquired from the same region, providing structural information and quantitative surface analysis within a unified framework [48].

Compared with conventional sequential characterization using independent instruments, SEM–AFM integration reduces sample transfer procedures, minimizes contamination risks, and improves spatial registration accuracy between different datasets. The elimination of laser alignment requirements through self-sensing cantilevers further simplifies system integration and enables stable operation in SEM-compatible environments [47]. As a result, SEM–AFM platforms provide a powerful approach for correlating nanoscale morphology with local mechanical, electrical, and interfacial properties.

### 2.2. Functional Capabilities of SEM–AFM

One of the fundamental capabilities of SEM–AFM is correlative nanoscale characterization, which relies on the complementary wide-field imaging and quantitative surface probing capabilities of the two techniques described in Section 2.1. By correlating SEM images with AFM measurements acquired from the same region, structural features observed by electron microscopy can be further quantified through AFM-derived topographical information, providing a more comprehensive description of nanoscale architectures than either technique alone [49,50].

A key advantage of SEM–AFM is the direct integration of AFM modules within SEM chambers, which enables a common coordinate framework between electron imaging and scanning probe measurements. Compact AFM modules can be incorporated into existing SEM platforms, with the AFM scan head positioned inside the SEM chamber to access the same region of interest observed by the electron beam [42]. Such integration minimizes the need for repeated sample transfer and improves spatial registration between SEM and AFM datasets. This capability is particularly valuable for heterogeneous materials, where nanoscale structural features identified by SEM require localized quantitative analysis using AFM.

Beyond topographical correlation, SEM–AFM provides a versatile platform for combining SEM-based structural localization with AFM-based functional measurements. Electrical AFM techniques, including C-AFM and KPFM, enable nanoscale mapping of current distribution, surface potential, and electronic heterogeneity. When combined with SEM-based structural information, these measurements allow local electrical properties to be correlated with specific microstructural features, such as defects, grain boundaries, and interfaces [51,52]. Similarly, AFM-based mechanical characterization methods, including force spectroscopy and nanomechanical mapping, provide quantitative information on elastic modulus, adhesion, deformation behavior, and frictional properties, enabling investigation of nanoscale structure–property relationships [53].

Recent developments in integrated SEM–AFM instrumentation have further advanced SEM–AFM toward multiparametric and correlative microscopy. By combining structural information obtained from SEM with functional measurements provided by AFM-based techniques, these platforms enable simultaneous or sequential investigation of morphological, mechanical, electrical, and interfacial properties within a unified experimental workflow. Consequently, SEM–AFM has evolved from a morphology-oriented imaging approach into a multifunctional characterization platform capable of linking local structures with material properties across multiple length scales [41].

For 2D materials, SEM–AFM correlation is particularly advantageous because the electronic, optical, and mechanical properties of these materials are highly sensitive to nanoscale structural and surface variations. SEM provides efficient large-area visualization and spatial localization, while AFM enables quantitative analysis of surface topography, thickness, and local physical properties. This complementary characterization strategy provides an effective experimental framework for establishing structure–property relationships in graphene, transition-metal dichalcogenides, and other atomically thin materials.

### 2.3. Comparison of Representative SEM–AFM Platforms and Conventional UHV SPM

Several commercial platforms now provide integrated or add-on SEM–AFM capabilities, and they differ significantly in the geometry between the AFM probe and the electron beam, which has direct consequences for imaging, force calibration, and measurement workflow. Three representative systems illustrate this diversity: the NenoVision LiteScope (NenoVision s.r.o., Brno, Czech Republic), the Quantum Design AFSEM nano (Quantum Design Microscopy GmbH, Pfungstadt, Germany), and the FusionScope (Quantum Design, Inc., San Diego, CA, USA).

The NenoVision LiteScope is an add-on AFM module that can be retrofitted into existing SEM chambers; its technology is marketed as correlative probe and electron microscopy (CPEM), in which the AFM probe is inserted at a fixed angle with respect to the sample while the electron beam retains access to the same region [41]. The tilted probe geometry allows the SEM to be used for navigation and for monitoring the probe, but the AFM scan is performed with the cantilever inclined with respect to the surface normal, which must be accounted for in force calibration and image interpretation.

The Quantum Design AFSEM nano is an AFM insert designed for SEM and FIB chambers, enabling atomic-force measurements to be combined with electron imaging and ion-beam processing within the same instrument. Its side-entry probe geometry is optimized for compatibility with FIB workflows, allowing, for example, site-specific cross-sectioning followed by AFM surface characterization.

FusionScope (Quantum Design) is a purpose-built combined SEM–AFM system in which the AFM and the electron column share a common sample stage, so that the same region can be measured with AFM functional modes (e.g., C-AFM, magnetic force microscopy, KPFM) and with SEM/EDS without moving the sample. Its geometry is optimized for rapid sequential or simultaneous access of the electron beam and the probe to the same area, at the cost of reduced flexibility compared with retrofittable modules.

In general, the probe–beam geometry determines a fundamental trade-off: configurations that give the electron beam unobstructed access to the sample (glancing or tilted probe) complicate quantitative force measurements and can shadow the beam, whereas configurations that optimize AFM performance (probe near the surface normal) constrain electron-beam access and the available working distance. The choice of platform therefore depends on the dominant requirement—navigation and correlative imaging versus quantitative AFM metrology—and on whether FIB or other in-chamber processing is required. Detailed technical specifications are available from the manufacturers, and a comprehensive technical assessment of combined AFM/SEM systems can be found in Ref. [41].

SEM–AFM can also be compared with conventional UHV AFM/STM. UHV scanning probe instruments provide the ultimate spatial resolution, including atomic-resolution imaging with STM and true atomic force measurements with qPlus sensors (quartz-based self-sensing force sensors), and are the method of choice for atomically resolved surface science, owing to their well-defined clean surfaces and low-noise environments. However, UHV systems offer only limited navigation capability, and large-area context is typically obtained by optical or low-magnification imaging rather than by electron microscopy. SEM–AFM platforms, in contrast, sacrifice the ultimate resolution and cleanliness of UHV operation but provide rapid electron-beam navigation, direct correlation between structural SEM and functional AFM data from the same region, in-chamber manipulation and processing capabilities, and access to multimodal measurements (e.g., SEM, EDS, FIB) that are not available in a UHV SPM environment. SEM–AFM is therefore most useful when the key requirement is correlative, multimodal, or in-situ characterization of heterogeneous samples at the micrometer-to-nanometer scale, whereas UHV AFM/STM remains preferable for atomic-resolution studies of clean, well-defined surfaces.

## 3. SEM–AFM and Correlative AFM Characterization of 2D Materials

2D materials exhibit diverse structural features across multiple length scales, including atomic defects, grain boundaries, wrinkles, layer-thickness variations, stacking configurations, and strain-induced distortions. For example, graphene serves as a fundamental building block for carbon-based materials of different dimensionalities, while more complex layered materials frequently exhibit heterogeneous structures arising from growth processes, interfacial interactions, and external stimuli [54], as illustrated in Figure 5. These structural characteristics strongly influence their electrical, optical, mechanical, and chemical properties, making comprehensive characterization essential for understanding structure–property relationships.

The increasing complexity of 2D material systems has imposed higher demands on characterization techniques. Conventional microscopy methods often provide only partial information regarding morphology, composition, or functionality, making it difficult to establish direct correlations between local structures and material properties. In contrast, SEM–AFM correlative platforms combine the rapid large-area imaging capability of SEM with the nanoscale topographical and functional mapping capability of AFM, enabling multidimensional characterization within a unified coordinate framework. Such integration facilitates the direct correlation of morphology, surface structure, and localized properties across multiple length scales. This correlative approach establishes a powerful “macro-screening to micro-analysis” characterization strategy that is particularly advantageous for low-dimensional materials with spatially heterogeneous properties. Building upon these advantages, SEM–AFM platforms have been increasingly employed to investigate the dynamic responses of 2D materials across multiple physical channels.

In the following sections, representative applications of SEM–AFM to the electrical, optical, mechanical, and in-situ multiphysics characterization of 2D materials are reviewed. According to the classification introduced in Section 1.3, most currently reported studies fall into the first category (sequential measurements), while genuinely integrated and truly in-situ platforms remain limited; this distinction is central to understanding the current state and future potential of SEM–AFM characterization.

### 3.1. Electrical Characterization of 2D Materials Through SEM–AFM Correlative Approaches

Electrical properties of 2D materials are highly sensitive to local structural heterogeneities. These features can locally modify conductivity, surface potential, carrier density, and charge transport, making it difficult to establish structure–electrical-property relationships using a single characterization technique. Following the classification introduced in Section 1.3, most studies discussed in this section belong to category (i), in which SEM and electrical AFM measurements are performed sequentially using independent instruments. In such workflows, SEM provides large-area structural localization, whereas electrical AFM modes, such as C-AFM and KPFM, provide nanoscale information on local conductivity and surface potential. Particular attention is therefore given here to defect-sensitive electrical AFM measurements, because grain boundaries and point defects often produce pronounced electrical heterogeneity even when their morphological signatures are weak.

A representative example of this sequential correlative strategy was recently reported by Limb et al. for bilayer 3R-MoS_2_ [55]. Scanning electron microscopy, C-AFM, and scanning electrochemical cell microscopy (SECCM) were applied to colocated regions of the same MoS_2_ crystal using separate measurements. C-AFM provided spatially resolved electronic-conductivity maps, while SEM revealed the morphology and layer distribution of the same crystal. Correlation with SECCM further showed layer-dependent electrochemical activity and nanoscale hotspots associated with defect-mediated processes. Importantly, SEM imaging was performed after the probe-based measurements, and the final SEM image revealed local surface damage introduced during C-AFM scanning. This example illustrates both the value and the practical limitation of sequential correlative measurements: complementary structural and electrical information can be obtained from the same region, but measurement order and probe-induced modification must be carefully considered.

Sequential SEM–AFM measurements are also useful for investigating electrically stimulated changes in 2D devices. Ansh et al. studied the degradation of chemical vapor deposition (CVD)-grown monolayer MoS2 field-effect transistors under prolonged electrical stress [56]. SEM imaging of a highly stressed device showed that the MoS_2_ channel remained physically continuous, whereas KPFM measurements of the same device revealed pronounced changes in surface-potential contrast. Grain boundaries and sulfur-related defects normally appeared as low-potential regions in KPFM, while electrically stressed channels exhibited unusual potential distributions associated with material reconfiguration and amorphization. The combination of SEM and KPFM therefore distinguished macroscopic structural continuity from local electrical degradation that could not be inferred from electron imaging alone. This study demonstrates how sequential structural and electrical measurements can reveal stimulus-induced changes in 2D electronic devices.

Defect-oriented studies further demonstrate the importance of electrical AFM modes in correlative characterization. Gentili et al. introduced sulfur-vacancy-rich patterns into MoS_2_ films and subsequently characterized their structural and electrical properties using SEM, KPFM, and peak-force tunneling AFM (PFTUNA) [57]. SEM revealed clear contrast between patterned and untreated regions associated with local desulfurization, whereas KPFM showed an increase in surface potential of approximately 12 mV within the vacancy-rich regions. Conductive PFTUNA mapping further revealed an increase of more than 30% in local conductivity compared with pristine regions. Notably, these electrically modified regions showed little contrast in conventional AFM topography, demonstrating that electrical AFM modes can reveal defect-related electronic variations that are not evident from surface morphology alone.

The defect sensitivity of electrical AFM is also supported by device-scale SPM studies. Minj et al. employed in-situ KPFM and scanning capacitance microscopy (SCM) to investigate the active channel of monolayer MoS_2_ field-effect transistors [58]. These measurements spatially identified electrically active grain boundaries, bilayer islands, and defective grain domains and revealed their contributions to gate-induced carrier modulation and channel conductance. Although this work did not employ SEM–AFM correlation and therefore does not belong to the sequential SEM–AFM category, it demonstrates the type of defect-specific electrical information that KPFM and SCM can contribute when incorporated into future correlative workflows.

Overall, these studies demonstrate the value of sequential SEM–AFM electrical characterization for 2D materials. SEM can rapidly locate device structures, layer boundaries, defects, and other regions of interest, while electrical AFM modes provide quantitative maps of conductivity, surface potential, and carrier-related variations at selected locations. This “large-area structural localization followed by localized electrical probing” strategy is particularly suitable for defect-sensitive and stimuli-responsive 2D materials. However, sample transfer, spatial relocation, environmental changes, and measurement-induced modification remain important limitations, and transient electrical responses cannot be directly captured by measurements performed at different stages. These limitations provide the motivation for the integrated and in-situ SEM–AFM approaches discussed in Section 3.4.

### 3.2. Optical Characterization of 2D Materials Through SEM–AFM Correlative Approaches

The optical responses of stimuli-responsive 2D materials are strongly affected by local structural heterogeneities, including grain boundaries, edges, folds, defects, interfaces, and strain fields. Because these features may occupy only tens of nanometers, conventional far-field Raman, PL, and infrared measurements often provide spatially averaged information. In a correlative SEM–AFM workflow, SEM can first identify structurally relevant regions over a relatively large field of view, while AFM and AFM-based optical techniques subsequently provide localized morphological, optical, and chemical information from selected regions. This sequential strategy is particularly useful for establishing structure–property relationships in 2D materials.

Tip-enhanced Raman spectroscopy (TERS), tip-enhanced photoluminescence (TEPL), and AFM-based infrared spectroscopy (AFM-IR) represent important extensions of AFM-based characterization for this purpose. TERS combines a plasmonic AFM tip with Raman spectroscopy to enhance the local electromagnetic field and achieve nanoscale vibrational imaging. TEPL similarly enhances localized photoluminescence emission and enables nanoscale mapping of excitonic behavior. AFM-IR detects local photothermal expansion induced by infrared absorption and therefore provides spatially resolved vibrational and chemical information [59,60,61,62]. These techniques are generally operated as standalone AFM-based platforms rather than as optical modules directly integrated inside an SEM chamber. Their present relevance to SEM–AFM characterization therefore lies mainly in sequential or same-region correlative workflows, in which SEM-based structural localization is followed by AFM-based functional measurements.

Representative experimental results illustrating the added value of these techniques are shown in Figure 6. For mono/bilayer WS2, TERS resolves monolayer–bilayer boundaries and local grain-boundary features that are less clearly distinguished in the corresponding diffraction-limited confocal Raman map (Figure 6a–c) [63]. The reported spatial resolution is approximately 40 nm, demonstrating the ability of TERS to reveal nanoscale vibrational heterogeneity that is averaged in conventional Raman measurements.

A similar improvement is observed for photoluminescence imaging. In a WSe_2_/Mo_0.5_W_0.5_Se_2_ heterobilayer, the TEPL map reveals nanoscale variations in interlayer-exciton emission that are not resolved in the corresponding conventional confocal PL image (Figure 6d,e) [64]. With a spatial resolution of approximately 20 nm, TEPL provides access to localized excitonic responses well below the diffraction limit. More generally, TEPL has also been used to investigate strain-induced bandgap modulation, edge-related excitonic states, and defect-associated luminescence in transition-metal dichalcogenides such as MoS_2_ and WSe_2_ [65].

AFM-IR provides complementary nanoscale chemical information. As shown in Figure 6f,g, SEM identifies the morphology of a PCNBS-functionalized reduced graphene oxide (rGO) flake, while the corresponding AFM-IR amplitude map acquired at 1220 cm^−1^ reveals the local infrared response associated with chemical functionalization [66]. This combined SEM and AFM-IR comparison illustrates the value of correlative characterization: SEM provides structural context, whereas AFM-IR adds spatially resolved chemical information that cannot be obtained from morphology alone.

**Figure 6:**
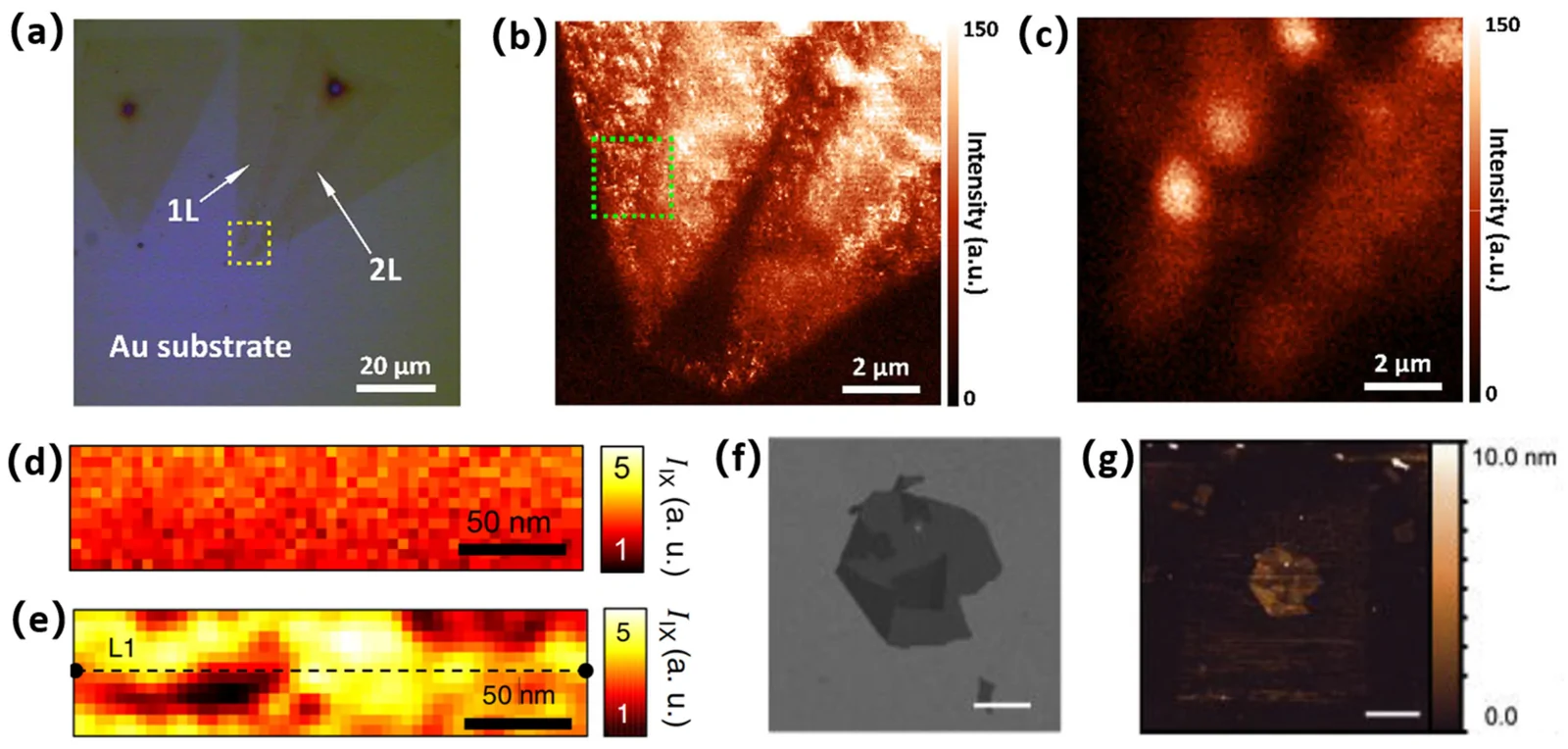
Representative experimental near-field optical characterization of 2D materials. (**a**–**c**) Mono/bilayer WS_2_: (**a**) optical image, (**b**) TERS A1g Raman map, and (**c**) corresponding confocal Raman map. Adapted from Lee et al. [63] under CC BY 4.0. (**d**,**e**) WSe_2_/Mo_0.5_W_0.5_Se_2_ heterobilayer: (**d**) confocal PL image and (**e**) corresponding TEPL map with ~20 nm spatial resolution. Adapted from Koo et al. [64] under CC BY 4.0; images were cropped and relabeled. (**f**,**g**) PCNBS-functionalized rGO: (**f**) SEM image and (**g**) corresponding AFM-IR amplitude map at 1220 cm^−1^. Adapted from Bartlam et al. [66] under CC BY 4.0; images were cropped and relabeled.

Conventional Raman and PL measurements have already been widely combined with electron microscopy to correlate optical responses with grain structures, defects, interfaces, and strain distributions in materials such as MoS_2_ and WS_2_ [67]. Near-field AFM-based techniques further extend this strategy by providing nanoscale vibrational, excitonic, and chemical information. In practical SEM–AFM workflows, SEM can therefore serve as a rapid structural screening and region-selection tool, followed by AFM, TERS, TEPL, or AFM-IR for localized functional characterization. Such sequential and same-region measurements represent an important intermediate stage between conventional independent characterization and future fully integrated in-situ multimodal SEM–AFM platforms.

### 3.3. Mechanical Characterization of 2D Materials Through SEM–AFM Correlative Approaches

Mechanical characterization of 2D materials through SEM–AFM correlative approaches combines the structural localization capability of SEM with the quantitative nanoscale probing capability of AFM-based mechanical techniques. AFM force spectroscopy, friction force microscopy, and nanoindentation have been widely employed to evaluate the elastic, adhesive, frictional, and fracture properties of atomically thin materials. In correlative workflows, SEM is particularly useful for identifying mechanically relevant structural regions, including suspended membranes, microfabricated cavities, wrinkles, grain boundaries, and heterointerfaces, thereby improving the positioning accuracy and reliability of subsequent AFM measurements.

Suspended graphene and TMD membranes are representative systems for AFM-based mechanical testing. Microfabricated substrates containing periodic hole arrays are commonly used to create freely suspended regions, whose geometry and integrity can be examined before mechanical loading. SEM can provide direct structural localization of these suspended areas, while AFM topography can further confirm membrane continuity and surface morphology prior to indentation [68]. In many benchmark studies, however, SEM localization and AFM nanoindentation are performed sequentially using separate instruments (category i), with spatial correlation relying on recognizable microhole arrays or other predefined structural markers.

AFM nanoindentation provides direct force–displacement responses from which elastic modulus, pretension, deformation behavior, and fracture strength can be extracted (the measurement geometry is shown schematically in Figure 7a). The suspended graphene membrane in Figure 7b is identified by optical microscopy rather than SEM, while the corresponding AFM nanoindentation response in Figure 7c provides a direct quantitative force–displacement relationship [69]. Although this example does not constitute an SEM–AFM measurement pair, it illustrates the type of mechanical data obtained after locating a suitable suspended membrane for AFM testing. Similar indentation measurements on monolayer graphene have reported a Young’s modulus of ~1 TPa and exceptional intrinsic strength associated with strong sp2 carbon bonding [70]. Related approaches have also been applied to suspended MoS_2_ membranes to investigate thickness-dependent deformation and fracture behavior [68].

In addition to point-wise indentation, AFM can provide spatially resolved nanomechanical maps over supported 2D materials. As shown in Figure 7d,e, AFM topography of monolayer MoS_2_ can be directly correlated with a corresponding in-plane Young’s modulus map acquired over the same region [71]. Such mapping distinguishes the mechanical response of the MoS_2_ layer from that of the underlying substrate and provides local information that cannot be obtained from topography alone. Compared with single-point force measurements, nanomechanical mapping is particularly useful for identifying spatial variations associated with thickness changes, defects, edges, interfaces, or local strain fields.

Beyond individual 2D crystals, SEM–AFM correlative mechanical characterization has also been extended to layered heterostructures. In graphene/h-BN systems, stacking configuration and interfacial registry can significantly influence adhesion, friction, and mechanical dissipation [72]. AFM-based friction measurements and force spectroscopy provide nanoscale information on these interfacial properties, while SEM assists in identifying the morphology and spatial distribution of heterostructure regions prior to mechanical analysis [73]. This combination of structural localization and quantitative mechanical probing helps connect local interface structure with mechanical response.

For environmentally sensitive 2D materials, correlative SEM and AFM measurements can further reveal how structural degradation influences mechanical behavior. Black phosphorus, for example, undergoes rapid morphological evolution under ambient exposure. SEM can reveal progressive structural changes, while AFM provides nanoscale surface information associated with degradation-related changes in the material state [74]. Such measurements are important for understanding the coupling between environmental stimuli, structural instability, and mechanical performance in air-sensitive 2D materials.

Overall, the mechanical information obtained from AFM extends SEM-based structural imaging from qualitative localization to quantitative nanoscale mechanics. Force–displacement curves provide local elastic and fracture information, whereas modulus, adhesion, or friction maps reveal spatial variations across supported or suspended regions. In current SEM–AFM workflows, these measurements are still predominantly sequential or same-region rather than truly simultaneous. Nevertheless, they provide an important foundation for the development of integrated in-situ SEM–AFM platforms capable of directly correlating structural evolution with mechanical response under external loading. Truly simultaneous mechanical testing with real-time electron-microscopy visualization has also been demonstrated using integrated in-situ SEM–AFM approaches, as exemplified by the work of Hummel et al. discussed in Section 3.4. Such platforms represent a further step beyond sequential and same-region measurements by enabling mechanical deformation and structural evolution to be monitored concurrently.

### 3.4. In-Situ and Stimuli-Responsive Characterization of 2D Materials Through SEM–AFM Platforms

Previous sections have demonstrated that sequential SEM–AFM approaches provide valuable information on the electrical, optical, and mechanical properties of 2D materials. However, such measurements generally rely on separate characterization stages before and after external stimulation, making it difficult to capture transient processes or localized evolution occurring during stimulus application. In-situ SEM–AFM platforms provide a solution by integrating electron microscopy and scanning probe measurements within a unified environment, enabling direct correlation between external stimuli and nanoscale material responses.

Compared with conventional SEM–AFM correlation, in-situ configurations maintain accurate spatial registration between SEM imaging and AFM probing while avoiding uncertainties caused by sample transfer and relocation. SEM provides rapid identification of structural features and morphological evolution, whereas AFM enables quantitative measurements of surface morphology, mechanical interactions, electrical properties, and local surface responses. Early studies demonstrated the feasibility of integrating AFM modules into SEM chambers for correlative nanoscale characterization [50]. Subsequent developments further improved system integration and expanded SEM–AFM platforms toward multiparametric analysis [41,75]. In addition, AFM-in-SEM approaches have been demonstrated as effective tools for comprehensive surface analysis by combining SEM-based localization with nanoscale AFM measurements within a single environment, further highlighting their advantages for correlated surface characterization [42].

For 2D materials, in-situ SEM–AFM is particularly attractive because the properties of these materials are strongly influenced by nanoscale structural variations. Such features may evolve rapidly under external perturbations and are difficult to relocate using sequential measurements. In-situ approaches therefore provide an effective strategy for investigating stimulus-induced structural and functional evolution in atomically thin materials. Figure 8 provides an example of same-region correlation within an integrated platform: SEM and AFM images of a graphene/h-BN heterostructure edge, acquired over the same region by AFM-in-SEM [76]; the direct correspondence between the SEM-identified edge and the AFM topography makes the spatial registration explicit. In addition, in-situ SEM/KPFM platforms have enabled correlation between structural features and local surface potential variations in semiconductor devices and 2D heterostructures [77].

Beyond passive observation, the development of stimulus-enabled SEM-based platforms has further expanded the capability of in-situ SEM–AFM toward dynamic characterization. AFM-based nanomanipulation systems integrated into SEM chambers have demonstrated that the probe can serve as both a measurement tool and a controllable mechanical actuator, enabling nanoscale manipulation with electron imaging feedback [78]. Meanwhile, microelectromechanical systems (MEMS)-based SEM holders incorporating heating and electrical biasing functions have provided important foundations for introducing controlled external conditions during electron microscopy characterization [79]. These advances establish the technical basis for investigating material responses under mechanical, thermal, and electrical stimuli.

Due to the atomic-scale thickness and high surface sensitivity of 2D materials, in-situ SEM–AFM offers unique advantages for directly linking applied stimuli with nanoscale structural and functional changes. Although fully integrated studies remain limited, several representative examples illustrating mechanical, electron-beam-induced, and electrical stimuli-responsive behaviors are summarized in Figure 9.

Mechanical deformation represents one of the most direct examples of stimuli-responsive behavior in 2D materials. Hummel et al. reported direct visualization of local deformation in suspended few-layer graphene membranes using a coupled in-situ SEM–AFM platform [80] (Figure 10). In Figure 10a, tapping-mode AFM reveals the three-dimensional morphology of a wrinkle in the suspended membrane, while the SEM image of the same region acquired at zero applied load (Figure 10b) allows the identical wrinkle to be identified by its electron-optical contrast; the direct correspondence between the two images illustrates the spatially registered topographical and structural information available within this platform. In this system, the AFM probe acted as a localized mechanical actuator, while SEM provided simultaneous high-resolution visualization of deformation processes in suspended graphene membranes. The integration of mechanical stimulation and real-time electron imaging enabled direct correlation between applied forces and nanoscale structural evolution, overcoming the limitation of conventional AFM mechanical measurements that mainly quantify local mechanical properties without directly capturing deformation dynamics. The real-time deformation of the same suspended membrane during loading is further documented in the original study [80] and its supplementary videos.

Electron-beam-induced modification provides another example of stimulus-driven evolution in 2D materials. Szkudlarek et al. investigated water-assisted etching of graphene induced by a focused electron beam and systematically studied the effects of electron-beam dose and dwell time on graphene removal and changes in surface morphology [81]. In this process, the electron beam acted not only as an imaging source but also as an external stimulus inducing localized structural modification. SEM was used to characterize the morphology and lateral dimensions of etched regions, while AFM provided quantitative analysis of the corresponding topographical profiles. The combined SEM–AFM correlative approach enabled comprehensive evaluation of electron-beam-induced graphene modification, demonstrating its potential for nanoscale processing and engineering of 2D materials. It should be noted that, in this example, the electron beam serves as a deliberately controlled stimulus; in other contexts, it constitutes an undesirable perturbation during imaging. This dual role of the electron beam is discussed further in Section 4.1.

In addition to structural responses, in-situ SEM–AFM-based approaches have also been extended toward functional characterization of 2D materials. In a further example of in-situ functional characterization, the correlative SEM/KPFM platform of Swain et al. [77] enables simultaneous acquisition of structural information and local surface potential distributions in semiconductor devices and 2D heterostructures. Although that study mainly focused on electrical characterization rather than dynamic structural evolution, it demonstrated the potential of in-situ SEM–AFM approaches for investigating functional responses associated with interfaces, defects, and charge redistribution in 2D heterostructures.

Although current examples of in-situ SEM–AFM-based stimuli-responsive characterization of 2D materials remain limited, these studies demonstrate the capability of this approach for investigating different response mechanisms, including mechanical deformation, electron-beam-induced modification, and electrical property variation. Future development of integrated platforms combining thermal control, electrical biasing, environmental regulation, and multimodal AFM sensing is expected to enable real-time investigation of more complex stimulus-induced processes in atomically thin materials. Furthermore, coupling multimodal SEM–AFM datasets with advanced data-driven analysis methods may provide new opportunities for understanding structure–property relationships in stimuli-responsive 2D materials. It should be kept in mind, however, that high-vacuum operation of SEM–AFM platforms restricts experiments involving gases, moisture, liquids, electrochemical environments, or chemically reactive components, as discussed in Section 4.2.

### 3.5. Summary and Classification of Representative Studies

To provide an overview of the current state of SEM–AFM characterization of 2D materials and to facilitate direct comparison between different experimental configurations, representative studies discussed in this review are summarized and compared in Table 1. Following the classification introduced in Section 1.3, the studies are grouped into three categories: (i) sequential SEM and AFM characterization using separate instruments, including SEM-guided workflows and correlative same-region analyses performed sequentially; (ii) integrated AFM-in-SEM platforms, in which the AFM module is installed inside the SEM chamber and the two measurements are performed sequentially within a common vacuum environment; and (iii) truly in-situ or simultaneous SEM–AFM measurements, in which electron imaging and AFM probing are performed concurrently on the same sample region, typically under an applied external stimulus. For each study, the table lists the material under investigation, the AFM mode, the SEM modality and external stimulus employed, whether acquisition was sequential or simultaneous, whether integrated or independent instrumentation was used, and the major advantages and limitations of the corresponding configuration.

Several observations can be drawn from Table 1. First, most reported studies on 2D materials fall into category (i): SEM is most frequently employed to identify structural features or to localize regions of interest, whereas quantitative AFM measurements—whether topographical, electrical, optical, or mechanical—are subsequently performed, often with sample transfer between instruments. This reflects the maturity and accessibility of sequential workflows, which do not require specialized integrated instrumentation. Second, integrated AFM-in-SEM platforms (category (ii)) are increasingly available and provide a common coordinate framework that eliminates sample transfer and improves spatial registration; however, most applications reported so far have focused on instrumentation development and general surface analysis rather than on stimuli-responsive 2D materials specifically. Third, truly in-situ or simultaneous SEM–AFM measurements under external stimuli (category (iii)) remain the least explored, with only a handful of representative examples, such as AFM-probe-induced mechanical loading of suspended graphene membranes [80] and in-situ SEM/KPFM measurements of 2D heterostructures [77]. These examples demonstrate the potential of genuinely integrated in-situ platforms for capturing dynamic structure–property evolution, but they also highlight that this capability is still at an early stage of development. Overall, Table 1 illustrates that the field is progressing from sequential approaches toward increasingly integrated and truly in-situ platforms, and it helps readers to distinguish solutions that have already been experimentally demonstrated from those that still present significant technical challenges, as discussed in Section 4.

## 4. Experimental Challenges and Limitations of SEM–AFM Characterization

While the preceding sections have focused on the capabilities of SEM–AFM characterization, a critical assessment of the associated experimental challenges is equally important, particularly for atomically thin and environmentally sensitive 2D materials.

### 4.1. Electron-Beam-Induced Effects

The integration of SEM with AFM necessarily exposes the sample to a focused electron beam, which can affect both the structure and the properties of atomically thin materials. Electron irradiation can induce local charging, especially in poorly conducting 2D materials and their insulating substrates, altering the effective surface potential measured by KPFM and perturbing other electrical measurements [77]. At higher doses, the beam can cause structural modification, including defect generation, amorphization, and electron-beam-induced etching or deposition—effects that may be exploited deliberately for nanoscale processing [81] but are undesirable in routine imaging. For environmentally sensitive materials such as black phosphorus, beam-induced damage can be difficult to distinguish from ambient degradation [74]. The electron beam can therefore act either as a controlled stimulus or as an unwanted perturbation depending on the experimental objective (Section 3.4). Experimental protocols should specify the intended role of the beam and, where possible, minimize adverse effects, for example by using low accelerating voltages, reduced dwell times, and minimized total electron dose.

The order of measurements is therefore an important practical consideration: when both SEM and KPFM (or other surface-sensitive electrical measurements) are required, AFM/KPFM should preferably be performed before SEM imaging, because electron irradiation can modify the surface potential and the work function, and such modifications are often irreversible; alternatively, low-dose imaging protocols should be adopted. Conversely, if electron-beam-induced changes are themselves the subject of the study (Section 3.4), SEM may deliberately precede the AFM measurements to monitor the induced evolution.

### 4.2. Contamination and Vacuum Effects

SEM–AFM measurements are generally performed under high vacuum, which offers both benefits and limitations. On the one hand, vacuum operation reduces capillary forces and facilitates contamination-free surface analysis; on the other hand, it restricts experiments involving gases, moisture, liquids, electrochemical environments, or chemically reactive components, and it can modify the intrinsic surface state of materials whose properties depend on adsorbates or hydration. Electron-beam-induced carbon contamination, arising from the cracking of residual hydrocarbons, can accumulate on the sample surface during imaging and degrade both SEM and subsequent AFM measurements. In addition, sample transfer between separate instruments in sequential workflows reintroduces ambient exposure, which is problematic for air-sensitive 2D materials and partially offsets the advantages of integrated platforms [42].

Electron-beam-induced contamination is of particular concern for surface-sensitive measurements: carbonaceous deposits formed during SEM imaging can locally modify the surface potential, reduce electrical conductivity, and alter frictional properties, thereby biasing subsequent KPFM, C-AFM, and friction measurements. Contamination control—through clean vacuum practices, plasma cleaning, and minimized beam exposure—is therefore essential when AFM functional measurements follow SEM imaging.

### 4.3. Instrumental and Experimental Limitations

Several instrumental factors limit the performance of SEM–AFM systems. Mechanical and thermal drift, which can be substantial inside the confined SEM chamber, degrade the spatial registration accuracy between electron images and AFM scans, particularly in long-duration experiments; drift correction and fiducial-based alignment are therefore essential for reliable same-region correlation [41]. The geometrical constraints of the SEM chamber restrict the size and orientation of AFM modules, limit the working distance available for electron imaging, and complicate simultaneous access of the electron beam and the probe to the sample. Probe performance is another consideration: self-sensing cantilevers avoid laser alignment but typically exhibit force sensitivity and noise characteristics different from those of optical beam-deflection systems [47], and probe wear during repeated contact-mode measurements affects data quality. Finally, because AFM is inherently a serial, point-by-point technique, the throughput of SEM–AFM characterization remains much lower than that of electron imaging alone; combined with the overhead of sequential workflows, this limits the statistical sampling achievable across large sample areas. These challenges are particularly relevant for atomically thin and environmentally sensitive materials, for which measurement conditions must be carefully controlled to obtain representative results.

A further limitation is the detection sensitivity of self-sensing cantilevers for very small features, which is an important challenge for future SEM–AFM systems: the integrated sensing elements generally have higher noise floors than OBD detection, and their sensitivity is coupled to the cantilever stiffness, making it difficult to simultaneously achieve high force sensitivity and high resonance frequency. This constrains the detection of sub-nanometer topographic features and weak electrical signals in atomically thin materials (see also Section 2.1).

### 4.4. Considerations for AFM Mode Selection on Fragile 2D Materials

The choice of AFM mode is a key practical consideration for fragile, atomically thin 2D materials, because different modes represent different trade-offs among force control, imaging speed, sample damage, and the ability to extract mechanical properties. Contact mode provides the highest lateral resolution and enables C-AFM and frictional measurements, but the continuous lateral tip–sample contact can damage weakly bound monolayers or displace adsorbates; it should therefore be used with a minimized loading force on 2D materials. Intermittent-contact (tapping/AC) modes greatly reduce lateral forces and are generally the default choice for imaging fragile 2D flakes and for determining layer number, but they cannot provide direct quantitative electrical contact and require careful setpoint control. Peak-force-based modes, in which the tip is modulated at low frequency and the peak force is held constant at each pixel, combine gentle imaging with the simultaneous extraction of force–distance curves, enabling modulus and adhesion mapping at controlled, low forces; they are well suited to suspended membranes and soft 2D systems. Non-contact modes minimize perturbation but provide limited topographical contrast and are rarely used for routine 2D characterization. For electrical measurements such as KPFM, lift-mode or amplitude-modulated implementations that avoid direct contact further reduce the risk of damage. In practice, the most suitable mode depends on the property of interest: low-force intermittent-contact or peak-force modes for topography and mechanical properties, contact or current-mapping modes for C-AFM, and non-contact or lift-mode KPFM for surface potential; in all cases, the minimum force required to obtain reliable data should be used.

## 5. Conclusions and Future Perspectives

The rapid development of stimuli-responsive 2D materials has highlighted the growing need for characterization techniques capable of simultaneously resolving structural, electrical, optical, mechanical, and environmental information across multiple length scales. By integrating the complementary strengths of SEM and AFM, SEM–AFM correlative microscopy has evolved into a powerful multimodal characterization platform that enables direct correlation between morphology, interfaces, defects, and local physical properties. This review has summarized recent developments in integrated SEM–AFM instrumentation, multimodal characterization methodologies, and representative applications in 2D materials, with emphasis on stimuli-responsive systems. Representative studies were classified into three progressively integrated configurations—sequential SEM and AFM characterization, integrated AFM-in-SEM platforms, and truly in-situ or simultaneous SEM–AFM measurements (Table 1)—and the experimental challenges and limitations of SEM–AFM integration, including electron-beam effects, contamination, vacuum constraints, and drift-related issues, were critically analyzed. Compared with single-technique characterization, SEM–AFM provides more comprehensive insights into complex physical processes by bridging large-area structural observation with nanoscale functional analysis. As instrumentation, multimodal integration, and intelligent data analysis continue to advance, SEM–AFM is expected to play an increasingly important role in the characterization, understanding, and development of next-generation low-dimensional functional materials.

Future SEM–AFM systems are expected to evolve beyond conventional correlative microscopy toward highly integrated multimodal characterization platforms. The incorporation of advanced electrical probing, nanomechanical mapping, scanning thermal microscopy, and near-field optical techniques within a unified instrument architecture will enable increasingly comprehensive investigations of dynamic material behaviors under coupled external stimuli.

A particularly promising direction lies in the development of intelligent SEM–AFM platforms. Recent advances in artificial intelligence have already demonstrated powerful capabilities in microscopy image analysis, automated feature recognition, and data-driven materials research. It should be emphasized that AI-assisted SEM–AFM characterization remains largely at the conceptual stage; the scenarios described below represent future directions rather than established capabilities. Realizing them will require addressing several practical issues, including image registration between SEM and AFM datasets with different fields of view and coordinate systems, the disparity in spatial resolution between electron imaging and probe-based measurements, the correlation of multimodal data with heterogeneous formats and noise characteristics, and the quantification of uncertainty in automated feature extraction and decision-making. Nevertheless, future integrated systems may benefit significantly from the incorporation of machine-learning-assisted experimental workflows. For example, SEM could be employed as a rapid screening tool to identify flakes, defects, interfaces, or stimulus-induced structural changes over large areas, while AI algorithms automatically determine regions of interest and guide subsequent AFM measurements. Such a hierarchical strategy could substantially improve characterization efficiency and reduce operator dependence.

Beyond automated feature selection, future AI-enabled SEM–AFM systems may establish closed-loop characterization workflows in which experimental decisions are continuously updated according to incoming data streams. Real-time analysis of SEM images, AFM topography, nanomechanical maps, and electrical measurements could enable adaptive adjustment of scanning parameters and autonomous exploration of complex material systems. For stimuli-responsive 2D materials, such systems may be capable of tracking dynamic processes including phase transitions, degradation, defect evolution, and interfacial reconstruction under electrical, optical, thermal, or environmental stimuli. More importantly, multimodal datasets generated by SEM–AFM could be integrated with materials informatics frameworks and predictive models, providing new opportunities for data-driven materials discovery and accelerated optimization of functional materials.

Another emerging possibility is the establishment of digital-twin characterization environments for nanoscale materials research. By combining correlative microscopy, real-time data fusion, and machine-learning prediction, future SEM–AFM platforms may not only characterize materials but also predict subsequent structural evolution and functional responses during experiments. Such capabilities could transform SEM–AFM from a passive observation tool into an active decision-making platform for autonomous materials research.

## Figures and Tables

**Figure 1:**
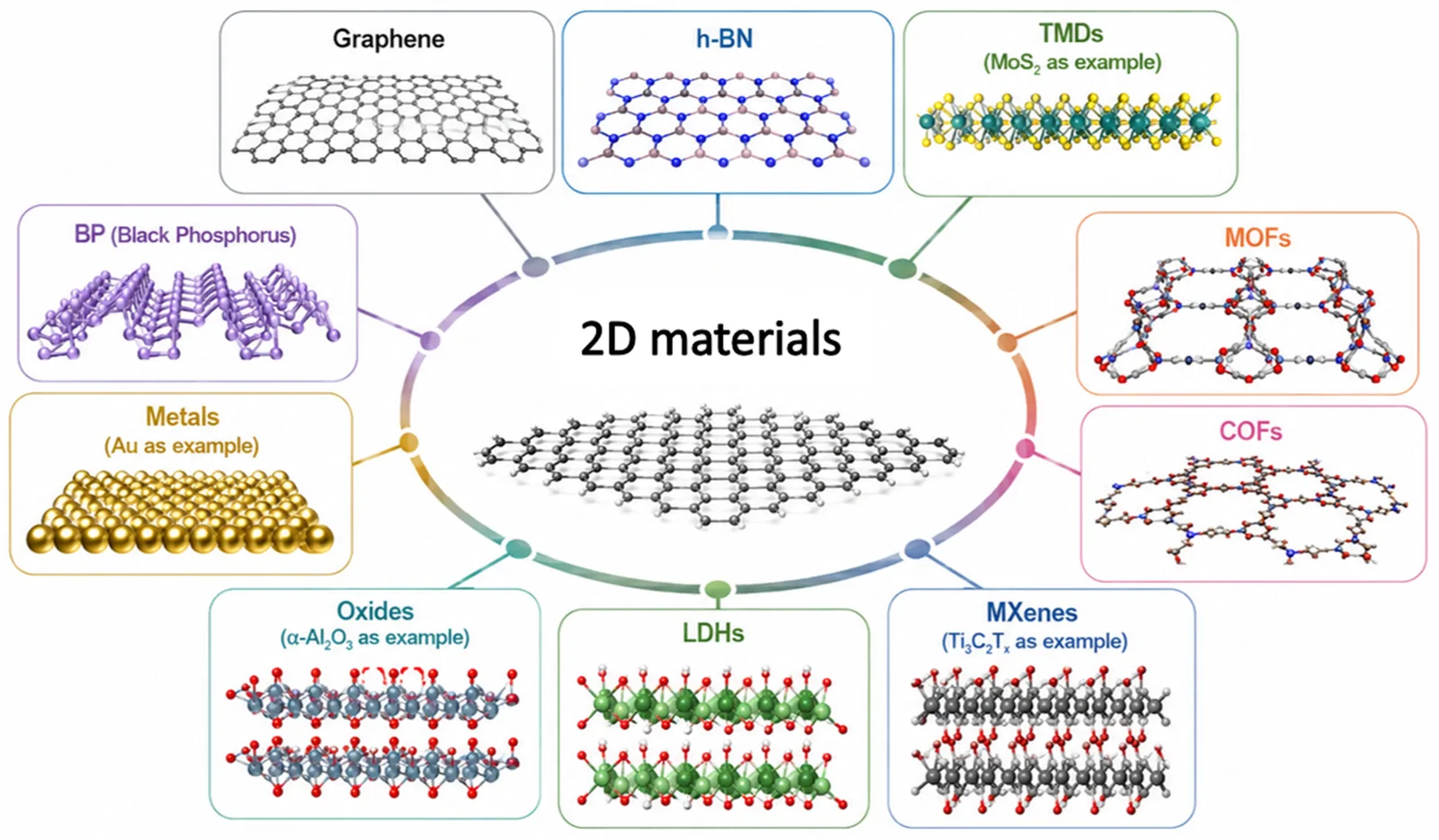
Schematic illustration of representative ultrathin 2D materials, including graphene, hexagonal boron nitride (h-BN), TMDs, metal–organic frameworks (MOFs), covalent organic frameworks (COFs), MXenes, layered double hydroxides (LDHs), oxides, metals, and BP. The representative atomic structures highlight the diverse chemical compositions, bonding configurations, and layered architectures of typical 2D materials.

**Figure 3:**
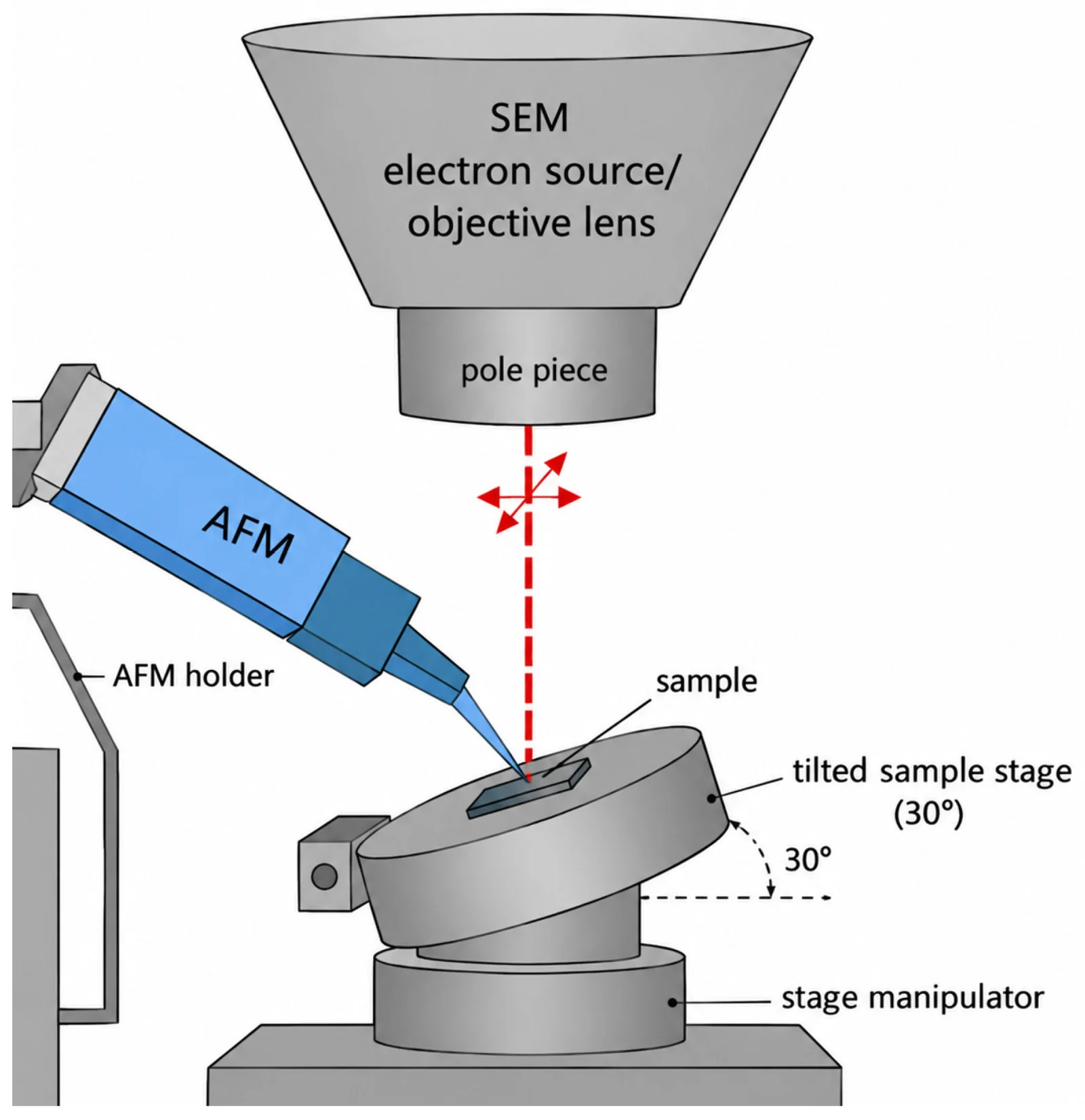
Schematic configuration of an integrated AFM-in-SEM system for correlative and in-situ characterization. The red dashed line represents the approximate trajectory of the SEM electron beam, which is emitted vertically downward. The arrows indicate that the electron beam can be deflected to a certain extent by the deflection field and act together with the AFM tip at different positions on the sample.

**Figure 4:**
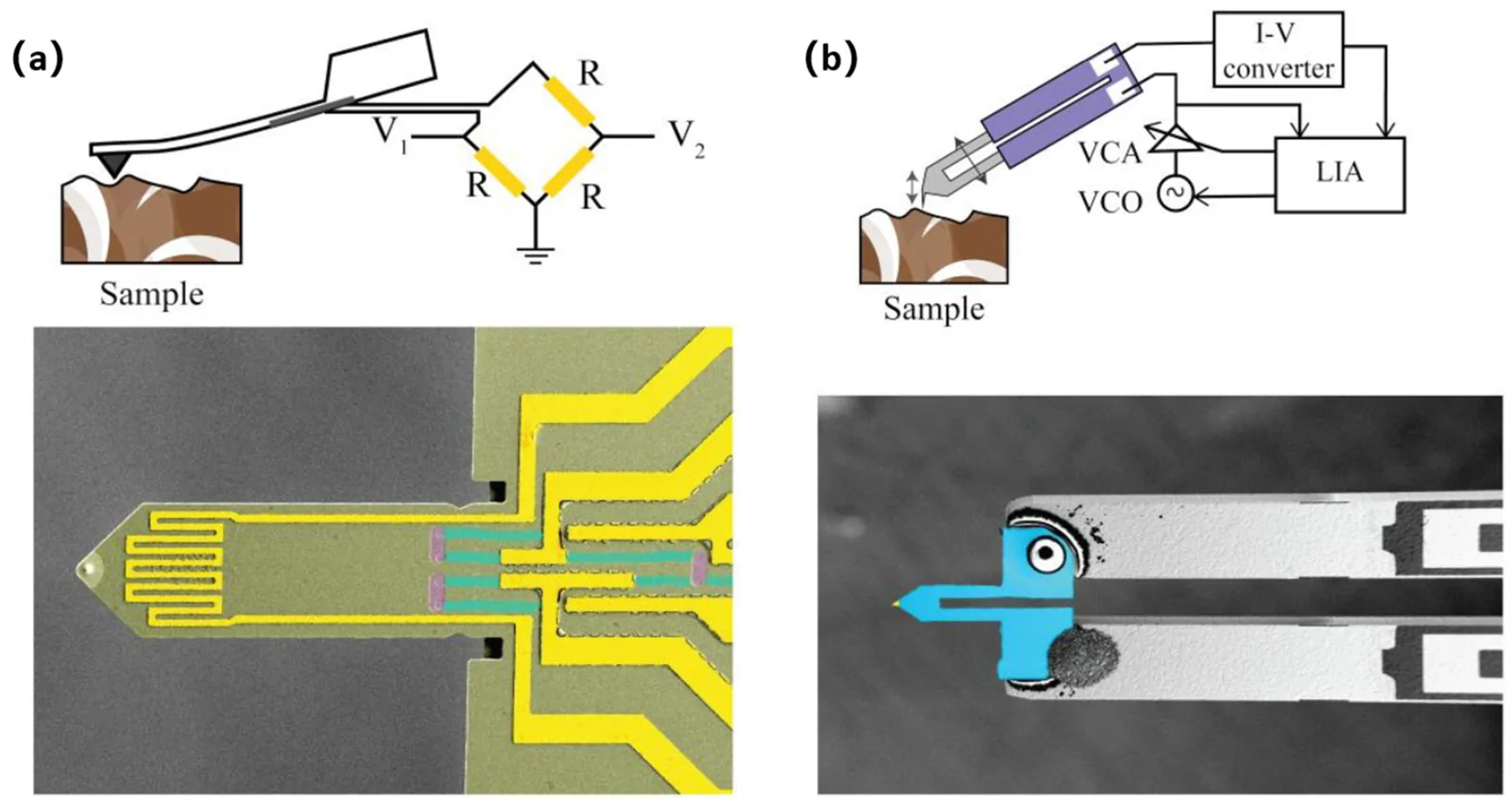
Schematic diagrams of two representative AFM sensing mechanisms integrated into SEM–AFM systems. (**a**) Schematic of a self-sensing piezoresistive AFM cantilever based on strain-gauge readout (**top**) and an SEM image of a representative piezoresistive cantilever with integrated electro-thermal actuators (**bottom**). (**b**) Schematic of an A-Probe-based AFM cantilever readout system (**top**) and an SEM image of a representative A-Probe cantilever (**bottom**). The lateral bidirectional arrow indicates the in-plane, anti-phase oscillation of the quartz tuning-fork prongs. This motion is mechanically coupled to the silicon cantilever and converted into an out-of-plane oscillation of the AFM tip, as indicated by the vertical arrow, enabling topographic sensing of the sample surface. Adapted from Swain et al. [41] under CC BY 4.0; images were cropped and relabeled.

**Figure 5:**
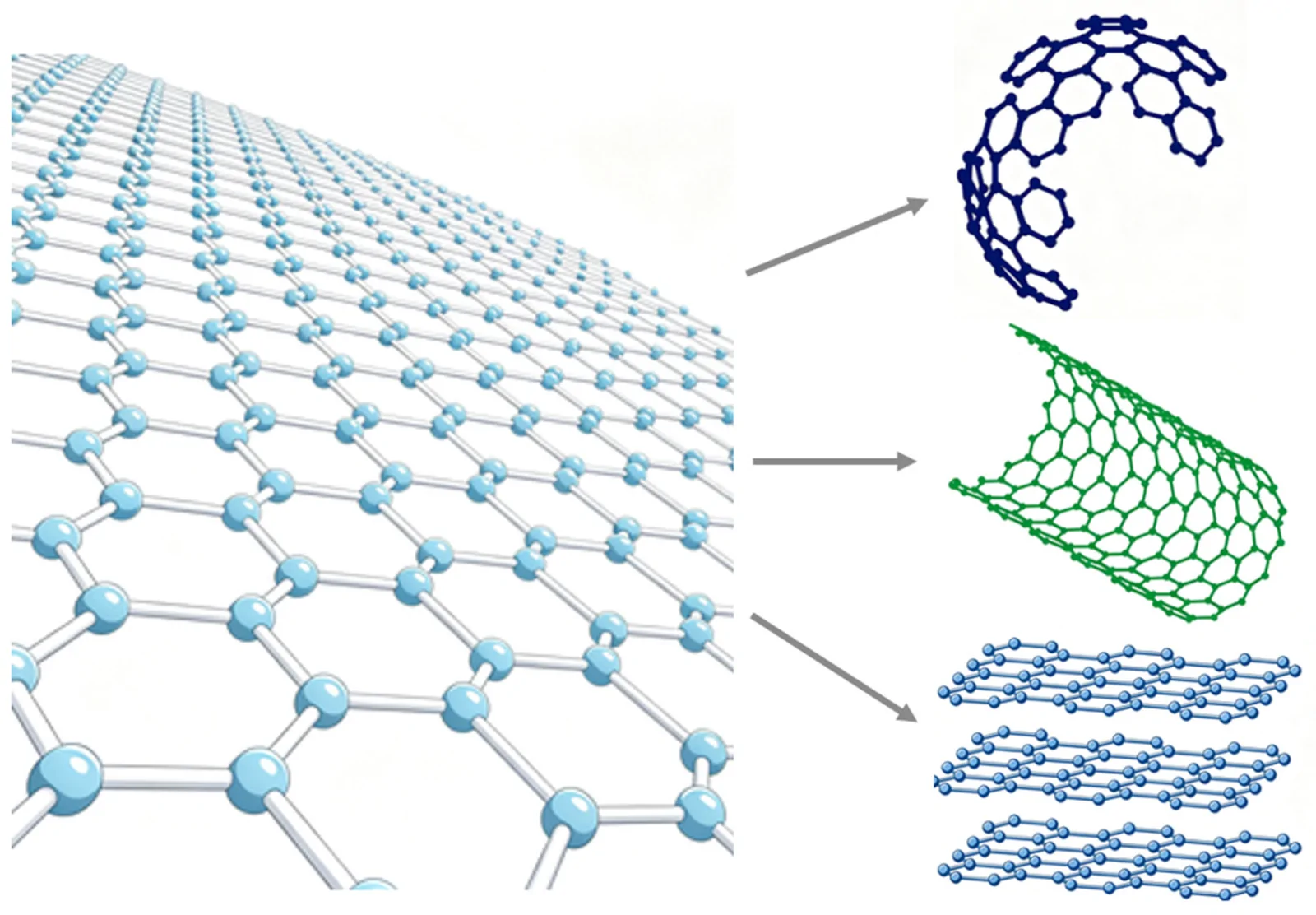
Graphene acts as a 2D building block for carbon materials across all other dimensions. It can be wrapped to form 0D buckyballs, rolled into 1D carbon nanotubes, or stacked to produce 3D graphite.

**Figure 7:**
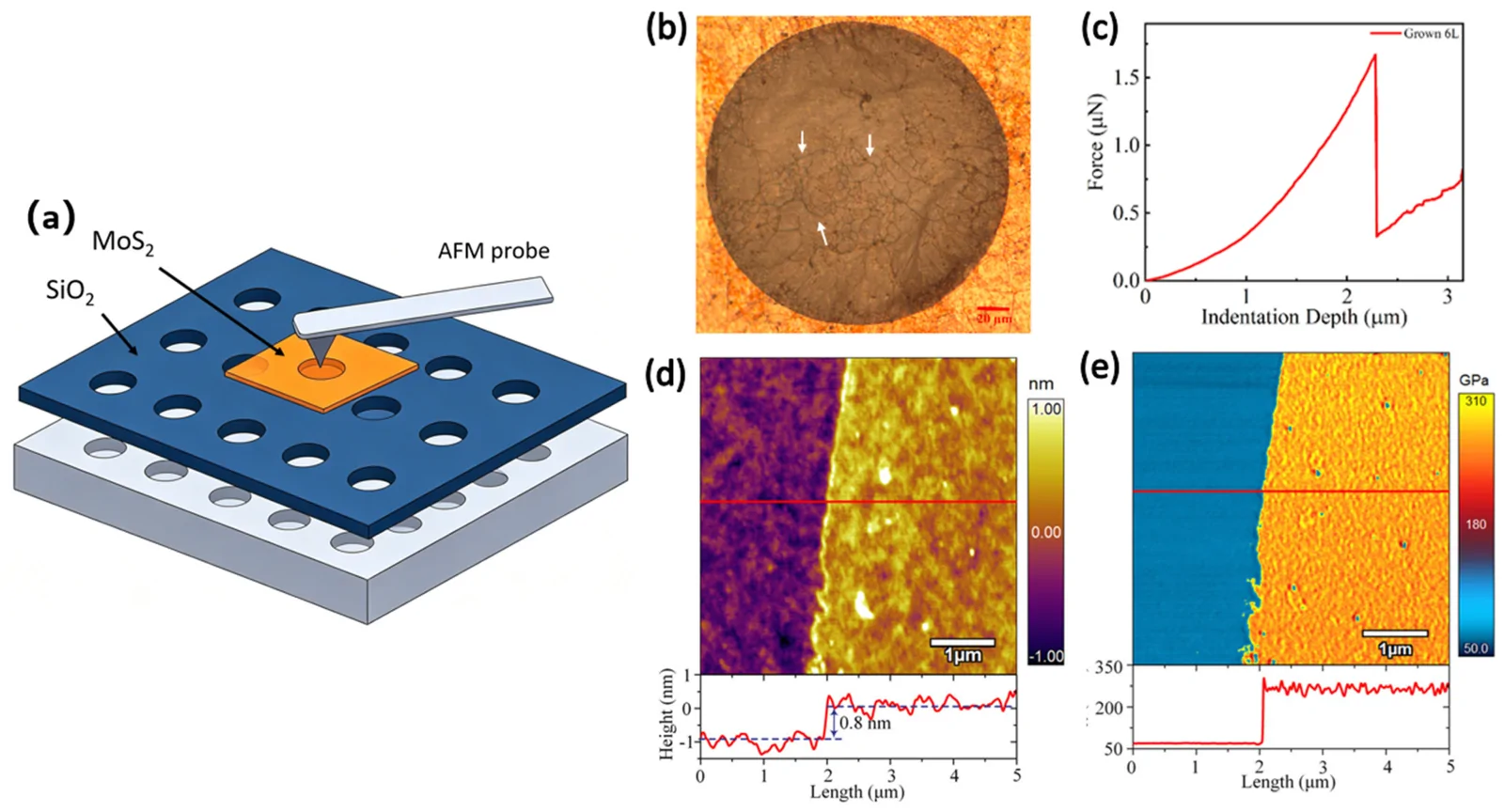
Representative mechanical characterization of 2D materials using AFM-based approaches. (**a**) Schematic illustration of AFM nanoindentation on a suspended 2D membrane. (**b**,**c**) Mechanical characterization of suspended graphene: (**b**) optical microscope image of a stacked suspended graphene membrane and (**c**) representative AFM nanoindentation force–displacement curve. Adapted from Xiao et al. [69] under CC BY 4.0; images were cropped and relabeled. (**d**,**e**) Nanomechanical mapping of supported monolayer MoS_2_: (**d**) AFM topography and (**e**) corresponding in-plane Young’s modulus map. Adapted from Li et al. [71] under CC BY 4.0; images were cropped and relabeled.

**Figure 8:**
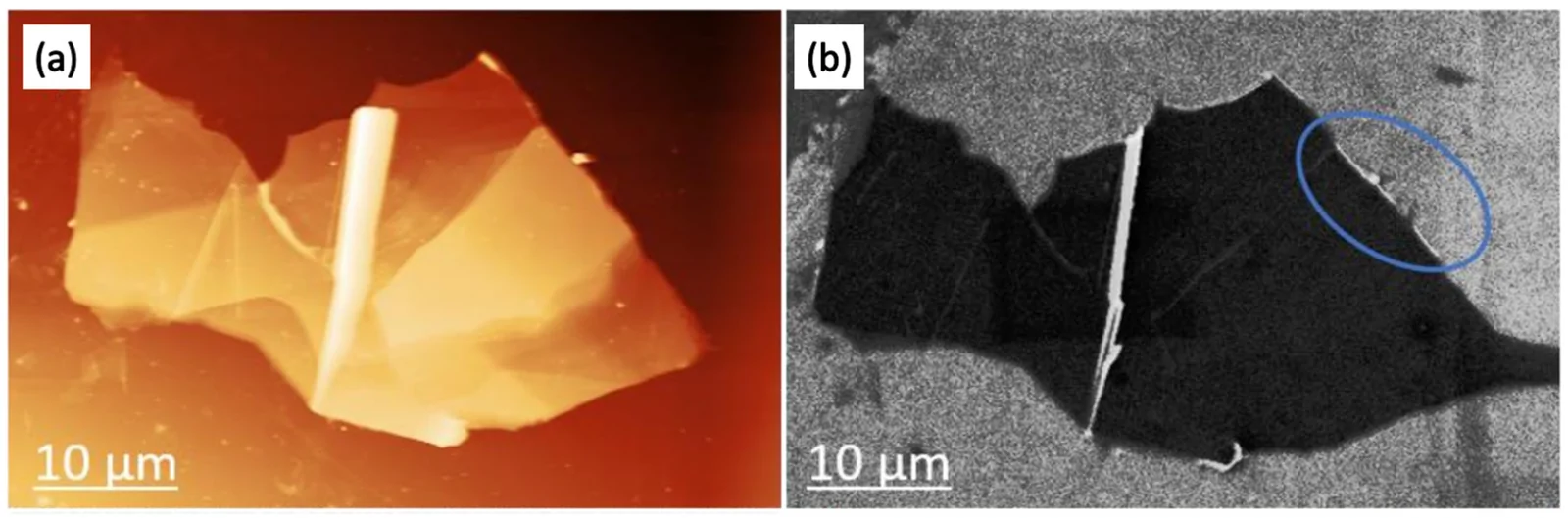
Correlative AFM-in-SEM imaging of a graphene/h-BN flake on a Si substrate: (**a**) AFM surface-height morphology and (**b**) SEM secondary-electron image (accelerating voltage 3 kV) of the same flake region, illustrating spatially registered structural and topographical information acquired within an integrated AFM-in-SEM platform. Adapted from Kuliček et al. [76] under CC BY 4.0; images were cropped and relabeled.

**Figure 9:**
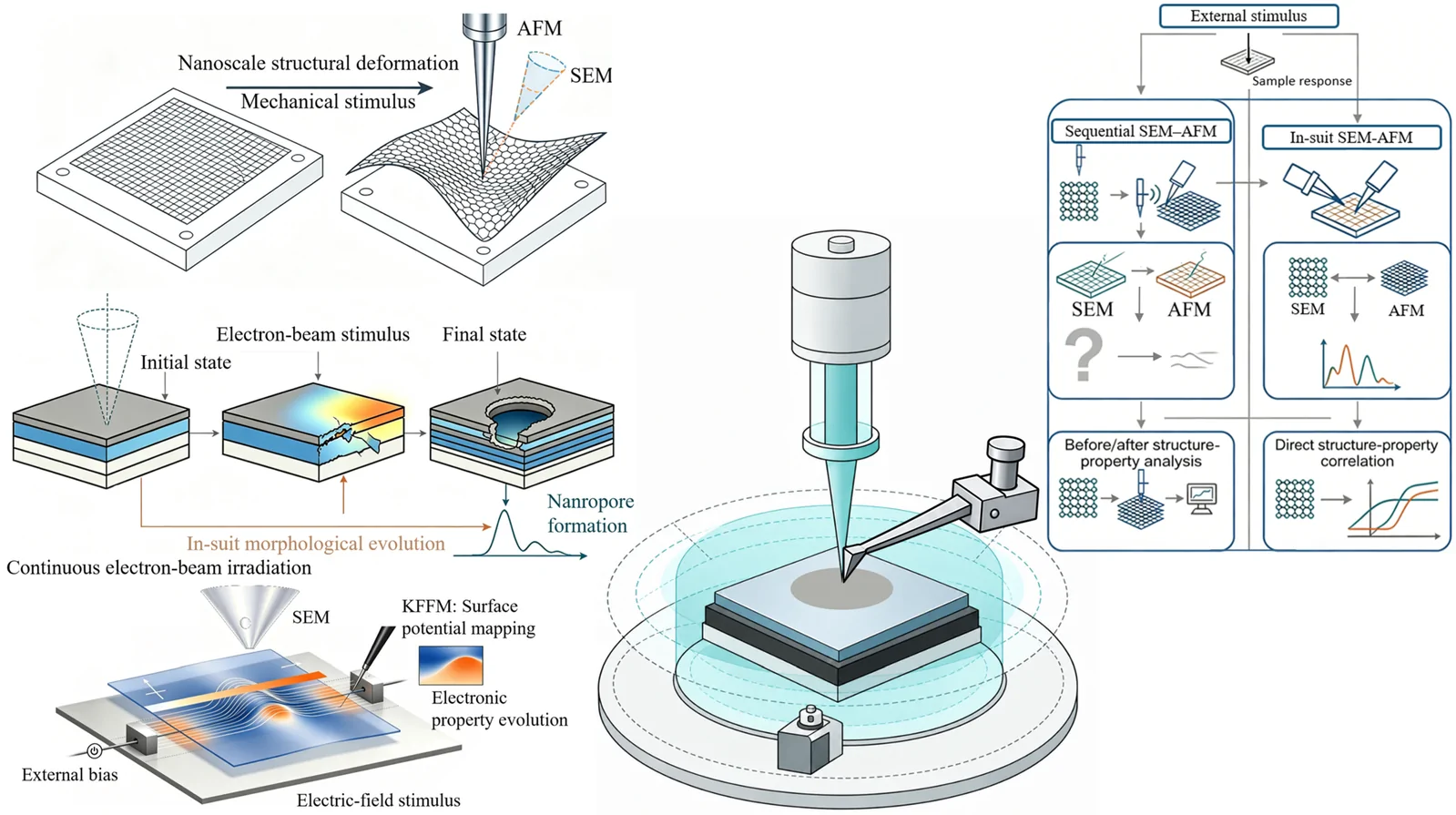
Schematic illustration of in-situ SEM–AFM platforms for dynamic characterization of stimuli-responsive 2D materials. The (**left**) panel summarizes representative stimulus-induced responses investigated by in-situ SEM–AFM, including mechanical deformation of graphene membranes under AFM probe loading, electron-beam-induced structural modification and nanopore formation, and electrically driven charge redistribution and surface potential variation in 2D heterostructures. The (**middle**) panel illustrates the integrated SEM–AFM configuration, combining electron-beam imaging with AFM-based nanoscale probing within a unified environment for simultaneous structural and functional characterization. The (**right**) panel compares conventional sequential SEM–AFM measurements with in-situ SEM–AFM approaches. Compared with sequential characterization that requires sample transfer and relocation before and after stimulation, in-situ SEM–AFM enables real-time monitoring of nanoscale structural evolution and functional responses, providing direct insight into structure–property correlations in stimuli-responsive 2D materials.

**Figure 10:**
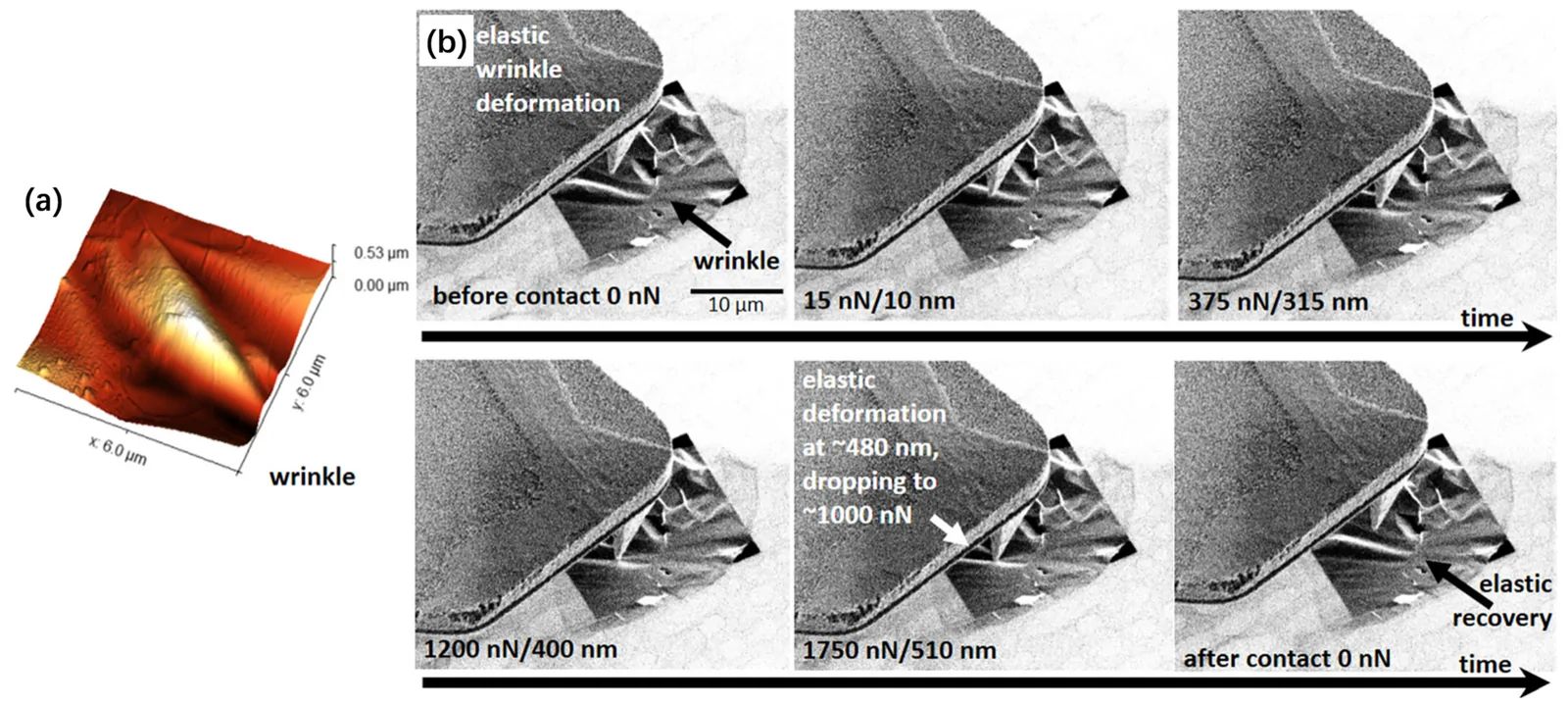
Correlative SEM–AFM imaging of a suspended few-layer graphene membrane: (**a**) three-dimensionally plotted tapping-mode AFM image of a wrinkle in the membrane and (**b**) SEM image of the same region at zero applied load, selected from a sequential SEM image series captured during AFM nanoindentation. The identical wrinkle is recognizable in both images, demonstrating spatially registered topographical AFM and structural SEM information. Adapted from Hummel et al. [80] under CC BY 4.0; images were cropped and relabeled.

**Table 1:** Summary of representative SEM–AFM platforms and characterization studies relevant to 2D materials, classified by measurement configuration (sequential, integrated AFM-in-SEM, and truly in-situ or simultaneous).

Category	Study (Ref.)	Material	AFM Mode	SEM Modality/Stimulus	Acquisition (Sequential/Simultaneous)	Instrumentation (Integrated/Independent)	Major Advantages and Limitations
(i) Sequential	Limb et al. [55]	Bilayer 3R-MoS_2_	C-AFM	SEM/SECCM electrochemical redox processes	Sequential, colocated	Independent instruments	Structural, electronic-conductivity, and electrochemical mapping of the same MoS_2_ crystal
(i) Sequential	Ansh et al. [56]	CVD-grown monolayer MoS2 field-effect transistor (FET)	KPFM	SEM/electrical stress	Sequential, same device	Independent instruments	Correlation of structural integrity with local surface-potential and defect-related changes after electrical stress
(i) Sequential	Bertolazzi et al. [68]	Monolayer MoS_2_	Nanoindentation	SEM localization of suspended regions/Mechanical loading	Sequential	Independent instruments	Quantitative elastic modulus and fracture behavior; ex situ, no real-time dynamics
(i) Sequential	Lee et al. [70]	Monolayer graphene	Nanoindentation	SEM localization of suspended regions/Mechanical loading	Sequential	Independent instruments	Young’s modulus ≈ 1 TPa and intrinsic strength; ex situ
(i) Sequential	Wood et al. [74]	BP	AFM topography	SEM imaging/ambient degradation	Sequential	Independent instruments	Tracking of degradation processes; difficult handling of air-sensitive samples
(i) Sequential	Szkudlarek et al. [81]	Graphene/SiO_2_	AFM topography	SEM imaging/electron-beam etching	Sequential	Independent instruments	Dose-controlled etching processing; risk of electron-beam damage
(i) Sequential	Garg et al. [67]	MoS_2_/WS_2_ heterostructure	Raman nano-imaging (TERS)	SEM localization/Raman excitation (optical)	Sequential	Independent instruments	Interfacial optical properties; not integrated
(i) Sequential	Bartlam et al. [66]	PCNBS-functionalized rGO	AFM-IR	SEM morphology/—	Sequential	Independent instruments	Nanoscale chemical functional-group identification combined with SEM structural context
(ii) Integrated platform	Kreith et al. [37]	Single-crystalline Cu_15_Zn brass	Tapping-mode AFM topography	SEM localization/nanoindentation	In-chamber sequential	Integrated (AFM-in-SEM)	Same-region SEM–AFM analysis in vacuum; quantitative slip-step-height and dislocation analysis
(ii) Integrated platform	Holz et al. [38]	General	AFM	SEM/—	In-chamber sequential	Integrated	Correlative microscopy and nanofabrication
(ii) Integrated platform	Alipour et al. [39]	General	AFM	SEM/—	In-chamber sequential	Integrated	Highly integrated platform
(ii) Integrated platform	Arat et al. [75]	General	AFM	SEM (+EDS)/—	In-chamber sequential	Integrated	AFM + SEM + EDS correlative analysis
(ii) Integrated platform	Novotna et al. [42]	General	AFM	SEM/—	In-chamber sequential	Integrated	Comprehensive surface analysis capability
(ii) Integrated platform	Kuliček et al. [76]	Graphene/h-BN	AFM	SEM/—	In-chamber sequential	Integrated	Heterostructure edge characterization
(iii) Truly in situ/simultaneous	Hummel et al. [80]	Suspended few-layer graphene	AFM probe as actuator	SEM simultaneous imaging/Mechanical deformation	Simultaneous	Integrated	Direct visualization of deformation; in-situ dynamics
(iii) Truly in situ/simultaneous	Swain et al. [77]	Semiconductor devices/2D heterostructures	KPFM	SEM/Electron beam, electrical	Simultaneous	Integrated	In-situ CPD imaging; preprint
(iii) Truly in situ/simultaneous	Mick et al. [78]	General	AFM nanomanipulation	SEM feedback/Mechanical	Simultaneous	Integrated	Probe manipulation with electron-beam feedback

## Data Availability

No new data were created or analyzed in this study. Data sharing is not applicable to this article.
